# Treatment of apicomarginal defect with periapical surgery: A case report

**DOI:** 10.4317/jced.57453

**Published:** 2020-11-01

**Authors:** David Peñarrocha-Oltra, Antonio Pallarés-Serrano, Pablo Glera-Suarez, David Soto-Peñaloza, Miguel Peñarrocha-Diago

**Affiliations:** 1 DDS, PhD. Assistant Professor, Oral Surgery Unit, Department of Stomatology, Faculty of Medicine and Dentistry, University of Valencia, Spain; 2 DDS, MSc. Master in Oral Surgery and Implant Dentistry, Department of Stomatology, Faculty of Medicine and Dentistry, University of Valencia, Spain; 3 DDS. Master in Oral Surgery and Implant Dentistry, Department of Stomatology, Faculty of Medicine and Dentistry, University of Valencia, Spain; 4 DDS, MSc, PhD. Master in Oral Surgery and Implant Dentistry, Department of Stomatology, Faculty of Medicine and Dentistry, University of Valencia, Spain; 5 MD, PhD, DDS. Full Professor, Oral Surgery Unit, Department of Stomatology, Faculty of Medicine and Dentistry, University of Valencia, Spain

## Abstract

An apicomarginal defect can be explained as a total loss of buccal alveolus extending from the original crestal bone to the apex of the tooth. This study presents a case of an apicomarginal defect in a first left molar subjected to periapical surgery with vestibular cortex block replacement and A-PRF + membrane coating approximately one year ago. One-year clinical follow-up was performed, with no evidence of recurrence. This case report discusses periapical surgical treatment and the importance of an interdisciplinary approach to the management of teeth with apicomarginal defects.

** Key words:**Periapical surgery, apicomarginal defect, A-PRF+, bone graft.

## Introduction

An apicomarginal defect can be explained as a total loss of buccal alveolus extending from the original crestal bone to the apex of the tooth (1). Apicomarginal defects constitute a significant challenge, since post-treatment long junctional epithelium formation occurs over the dehisced root surface (2). The amount and location of bone adjacent to the root structures affect the prognosis of periradicular surgery. An apicomarginal defect has an adverse effect upon the outcome, reducing the complete healing rate (3), since healing takes place by repair rather than regeneration (4).

The role of platelet-rich fibrin (PRF) has been well documented in regeneration processes for the management of periodontal intrabony defects, gingival recessions and furcation defects (5). Advanced platelet-rich fibrin (A-PRF) is a low-speed centrifugation concept protocol (6). Within the A-PRF family, A-PRF+ demonstrates significantly higher levels of messenger RNA (mRNA) encoding for growth factors and type 1 collagen (6). The A-PRF+ matrix is characterized by a more porous structure, favoring vascularization and cell penetration, as well as the homogeneous distribution of platelet-derived growth factors and leukocytes (7). The abovementioned aspects encourage the adoption of alternatives capable of enhancing wound healing, like the use of A-PRF+ membranes.

## Case Report

**Figure 1:**
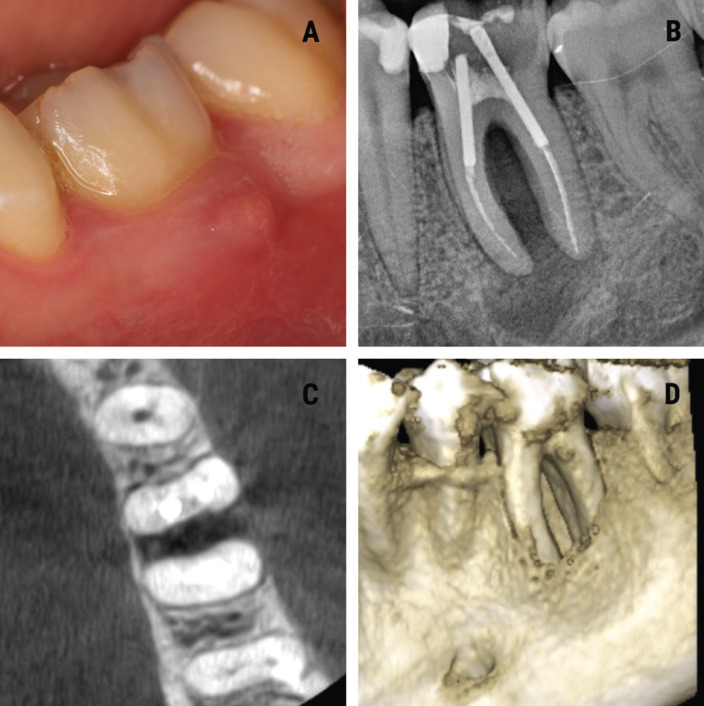
A. Intraoral view showing an abscess in the attached gingiva at level 3.6. B. Periapical radiograph showing a radiolucency along the roots of 3.6. C. Tomographic (CBCT) axial sectional view where destruction of the vestibular cortical layer and involvement of the bone around the furrow of 3.6 can be seen. D. Three-dimensional tomographic (CBCT) reconstruction showing the bone lesion around the entire root of tooth 3.6.

A 29-year-old systemically healthy non-smoking female reported to the Department of Oral Surgery of the University of Valencia (Valencia, Spain) with mild pain and purulent discharge in relation to the lower left posterior tooth region during the last month. At intraoral examination, the gingival tissue attached to the vestibular surface of the first lower left molar showed the presence of an abscess (Fig. 1A). The patient reported no history of trauma to the tooth. The first lower left molar had undergone root canal treatment three years ago. From the periodontal perspective, the dentition was sTable and clinically healthy, but tooth 3.6. presented a localized deep probing depth of 9 mm along the vestibular surface of the mesial root. Intraoral periapical radiography of the affected tooth revealed the presence of bone destruction around the roots of the lower molar (Fig. 1B). An ill-defined radiolucency was observed surrounding the three-thirds of the roots, and compromising the molar furcation.

Cone-beam computed tomography (CBCT)(Planmeca®, Helsinki, Finland) revealed the presence of localized periapical bone loss with loss of vestibular bone cortex and marginal alveolar bone around the mesial root, compromising the furrow of molar 3.6. (Fig. 1C,D). Based on the CBCT findings, a diagnosis of apicomarginal bone defect was established. The clinical and radiographic examination confirmed persistent chronic apical periodontitis caused by lower left first molar 3.6.

The patient signed the informed consent to treatment and the use of data for scientific purposes. Periapical surgery was performed, with regeneration of the apicomarginal defect with vestibular cortical block autograft and A-PRF + membrane coating.

For A-PRF+ preparation, the median cubital vein was punctured to collect three 10-ml glass tubes without additives (Plain Vacuum Tube, A-PRFTM+, Process for PRF, Nice, France). The tubes were centrifuged at 1300 rpm for 8 min (A-PRFTM DUO centrifuge, Process for PRF, Nice, France). The fibrin clots obtained were extracted with special tweezers and scissors, avoiding the red blood component below the buffy coat, and were further processed to obtain a standardized membrane thickness in a sterile container (PRF-BOX, Process for PRF, Nice, France).

**Figure 2:**
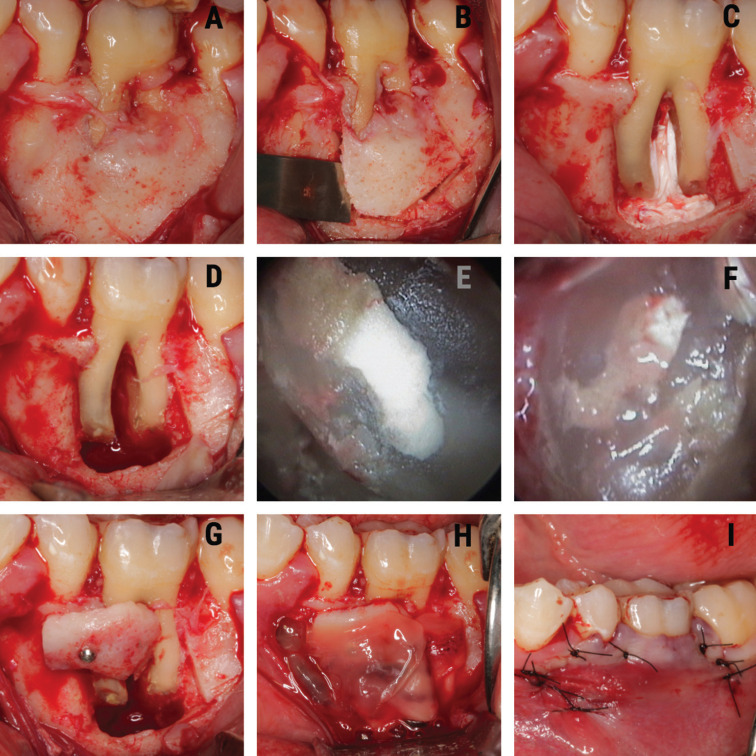
A. Intraoperative view showing involvement of the vestibular cortex of the mesial root of 3.6. B. Collection of the autograft in vestibular cortex block. C. Apicoectomy, performing the retrograde cavity in the mesial and distal roots of 3.6. and placement of polytetrafluoroethylene strips. D. Retrograde obturation with MTA in the mesial and distal roots. E. Fixation of the autograft in vestibular cortical block repositioned in the coronal third of the mesial root of 3.6. F. Bone defect coating with A-PRF + membranes. G. Postoperative view of the simple sutures. H. Postoperative radiographic view.

Periapical surgery began with anesthetic infiltration at the bottom of the vestibule of 3.6. using two 4% articaine carpules with 1: 100,000 epinephrine (Inibsa®, Lliça de Vall, Barcelona, Spain). A sulcular incision was performed, with mesial release of 3.5 and another distal to 3.7., with raising of a full thickness trapezoidal flap (Fig. 2A).

Ostectomy of the vestibular cortex was performed with ultrasound tips (Piezomed®, W&H, Bürmoos, Austria), and a vestibular cortex block with was obtained chisel and hammer (Fig. 2B).

The margins of the vestibular bone cortex lesion were profiled with a tungsten carbide burr mounted on a handpiece with abundant irrigation of physiological saline solution, allowing visualization of the entire lesion. Then, the periapical inflammatory tissue was removed and the apicoectomies of the two roots of the lower left first molar were performed.

Once root integrity of the first lower left molar was checked, the retrograde obturation box was performed with ultrasound tips (Piezomed®, W&H, Bürmoos, Austria). After proper control of hemostasis with polytetrafluoroethylene strips (Fig. 2C), it was retrogradely sealed with mineral trioxide aggregate (MTA) (ProRoot®, Dentsply, PA, USA). The MTA was compacted in the retrograde cavity and, after waiting 7 minutes for initial setting, it was polished with a red ring burr (Fig. 2D). Retrograde filling of the two mesial root canals (Figure 2E) and distal root canal (Fig. 2F) was checked with an endoscope (Karl Storz®, Tuttlingen, Germany) (Fig. 2E,F).

Regeneration of the apicomarginal defect was performed with replacement of the vestibular cortex, fixed in the coronal third of the tooth root, which was fixed with a screw (Bone Management® System Master-Pin-Control, Meisinger, USA) (Fig. 2G), and the buccal cortical autograft and bone defect were covered with A-PRF + membranes (Fig. 2H).

Suturing was performed with non-reabsorbable 5/0 multifilament suture (Tevdek®, Teleflex®, PA, USA) (Fig. 2I). The patient was prescribed 500 mg of amoxicillin three times a day for 7 days, 600 mg of ibuprofen three times a day for three days, and 0.12% chlorhexidine rinses during 7 days. The sutures were removed one week later.

**Figure 3:**
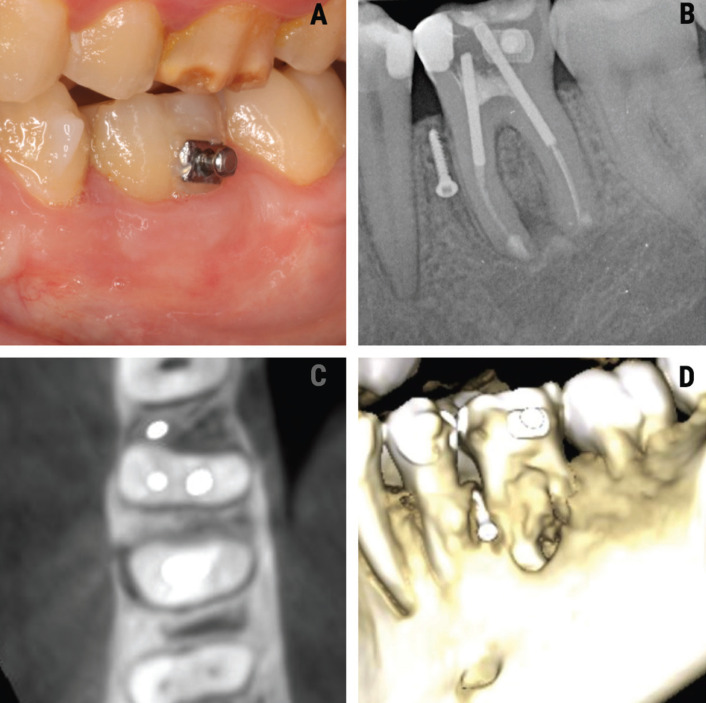
A. Intraoral view of tooth 3.6 after one year. B. Periapical radiograph showing bone trabeculation between the roots of the lower left first molar. C. Tomographic view showing the formation of bone around the furrow of 3.6. one year after periapical surgery. D. Three-dimensional tomographic (CBCT) reconstruction of the current condition of tooth 3.6.

One year after surgery, the patient underwent clinical (Fig. 3A) and radiological evaluation (Fig. 3B). There were no clinical symptoms, and the tomographic study showed radiological healing of the lesion together with the formation of bone around the furcation of the lower left first molar (Fig. 3C,D).

## Discussion

Of the present case corresponds to a patient with an extensive apicomarginal bony defect. Treatment included the elimination of endodontic infection with periapical surgery, a vestibular cortex block autograft, and its coating with A-PRF membranes. The occurrence of periapical disease, accompanied by periodontal breakdown, constitutes a complex problem in periradicular surgery - being typically associated with a less favorable prognosis, since disruption of the cortical plate can have a deleterious effect upon regeneration of the lost tissues (8,9). Destruction of marginal bone found in apicomarginal defects reduces the success rate of treatment to 27% and 37% (10).

The regeneration of periapical tissues after periapical surgery requires complex interactions among recruited stem cells to secure differentiation into committed osteoblasts, periodontal ligament cells and cementoblasts (11). Growth factors are necessary signals for the attachment, migration, proliferation and differentiation of the stem cells, and local microenvironmental cues, such as adhesion molecules are also important, as well as an extracellular matrix and associated non-collagenous protein molecules (12). The lack of any of these elements results in tissue repair rather than regeneration (4).

The present case report highlights the role of PRF in the healing of apicomarginal defects in the mandibular first molar sector. It has been observed that intrinsic growth factors (the host’s own biological products) are better in promoting wound healing than extrinsic growth factors (13). Platelet-rich fibrin is an osseoinductive material that enhances osteogenesis in comparison with the physiological healing process (14). The PRF membrane also acts as a barrier, accelerating wound closure and mucosal healing due to fibrin bondage and growth factor release (15). For this reason we decided to use PRF as a source of growth factors.

Healing in our case could be attributed to three factors: the immune regulatory action of PRF; the vestibular cortical block autograft repositioned in the coronal third of the mesial root of the lower left first molar; and the removal of infection from the surgical site, promoting connective tissue and bone formation from adjacent healthy periodontium (12).
